# Determine independent gut microbiota-diseases association by eliminating the effects of human lifestyle factors

**DOI:** 10.1186/s12866-021-02414-9

**Published:** 2022-01-03

**Authors:** Congmin Zhu, Xin Wang, Jianchu Li, Rui Jiang, Hui Chen, Ting Chen, Yuqing Yang

**Affiliations:** 1 School of Biomedical Engineering, Capital Medical University, Beijing, China; 2 Beijing Key Laboratory of Fundamental Research on Biomechanics in Clinical Application, Capital Medical University, Beijing, China; 3 Institute for Artificial Intelligence and Department of Computer Science and Technology, Tsinghua University, Beijing, China; 4 Department of Ultrasound, Peking Union Medical College Hospital, Beijing, China; 5 Bioinformatics Division and Center for Synthetic & Systems Biology, Beijing National Research Center for Information Science and Technology, Department of Automation, Tsinghua University, Beijing, China; 6 State Key Laboratory of Networking and Switching Technology, Beijing University of Posts and Telecommunications, Beijing, China

**Keywords:** Gut microbiota, Human variables, Disease classification, Machine learning

## Abstract

Lifestyle and physiological variables on human disease risk have been revealed to be mediated by gut microbiota. Low concordance between case-control studies for detecting disease-associated microbe existed due to limited sample size and population-wide bias in lifestyle and physiological variables. To infer gut microbiota-disease associations accurately, we propose to build machine learning models by including both human variables and gut microbiota. When the model’s performance with both gut microbiota and human variables is better than the model with just human variables, the independent gut microbiota -disease associations will be confirmed. By building models on the American Gut Project dataset, we found that gut microbiota showed distinct association strengths with different diseases. Adding gut microbiota into human variables enhanced the classification performance of IBD significantly; independent associations between occurrence information of gut microbiota and irritable bowel syndrome, *C. difficile* infection, and unhealthy status were found; adding gut microbiota showed no improvement on models’ performance for diabetes, small intestinal bacterial overgrowth, lactose intolerance, cardiovascular disease. Our results suggested that although gut microbiota was reported to be associated with many diseases, a considerable proportion of these associations may be very weak. We proposed a list of microbes as biomarkers to classify IBD and unhealthy status. Further functional investigations of these microbes will improve understanding of the molecular mechanism of human diseases.

**Supplementary Information:**

The online version contains supplementary material available at 10.1186/s12866-021-02414-9.

## Introduction

The human intestines are home to a dense microbial community, collectively known as the gut microbiota [1]. The gut microbiota forms a complex ecosystem and performs a wide range of functions with far-reaching impacts on human health, including extracting energy from the digestive system, preventing colonization by pathogens, promoting immune homeostasis, producing important metabolites, and even communicating with the central nervous system via the gut-brain axis [2]. So, it is thought to play an important role in the development of many diseases, including inflammatory bowel disease [3], *Clostridium difficile* infection [4], diabetes [5], cardiovascular disease [6], and mental health disorders [7]. The gut microbiota determines certain host characteristics and responds to host variables, such as human lifestyle and physiological variables, which can be reflected in the microbial composition [8]. Therefore, a considerable part of the human variables on human health and disease risk may be mediated or modified by gut microbiota.

With the development of high-throughput sequencing technology, we are now able to sequence the hypervariable regions of the 16S rRNA gene and cluster into operational taxonomic units (OTUs) to profile the taxonomic composition of the microbial community in an environmental sample [9]. Over the last few years, many case-control studies have been conducted to collect microbial 16S rRNA gene datasets from human fecal samples to explore the associations among the gut microbial community and human diseases to reveal disease-specific microbial biomarkers [10, 11]. However, many investigations showed low concordance on the discovered disease-associated microbes and one obvious example is about obesity. Gut microbiota reported by multiple studies of which abundance is differential between obese and lean individuals is inconsistent [12]. Furthermore, Sze and Schloss [13] comprehensively analyzed the results of several obesity-related studies. They found that the statistical detection power of a small-sample study was insufficient, and the ratio of abundance of *Bacteroidetes* and *Firmicutes* was not associated with obesity. In addition, recent construction of a large dataset from the Swedish population did not reveal an apparent microbial signature associated with irritable bowel syndrome (IBS) as previously reported in the literature, and the heterogeneity of the microbial community among IBS patients was higher than that among healthy individuals [14].

There are two possible reasons which may result in low concordance in previous studies. One is the limitation of the sample size. Generally, there are thousands of microorganisms with a wide range of abundance levels in intestinal samples. Due to the high cost of building a large-scale dataset consisting of both gut microbiota information and elaborate human variables [15], researchers can only afford to sequence dozens or hundreds of samples to explore disease-associated microbes via statistical models. Thus, the model overfitting is common and thus reduces the reliability of the inferred results. The other critical shortcoming is neglecting the influence of host variables, which makes it difficult for researchers to confirm whether the calculated gut microbial-disease associations indicate the true interactions between microbes and the progression of diseases. The alternative possibility is that microbes are only related to certain host variables, and as a result, they are associated indirectly with diseases [16].

Therefore, a large-scale dataset containing information on both gut microbial community and host variables is required for the accurate identification of microbiota-disease associations. Fortunately, the American Gut Project (AGP), which comprised thousands of 16 s rRNA gene sequencing samples and a rich human variables set related to human lifestyle and physiological variables and diseases, has been carried out worldwide [17]. Today, the AGP has sequenced more than 15,000 samples, which significantly expands human gut microbiota’s existing data. Most importantly, it provides a rich resource for each sample with information on gut microbiota, human lifestyle factors, and diseases. The goal of this study is to explore the relationship between these entities using this dataset.

**Fig. 1:**
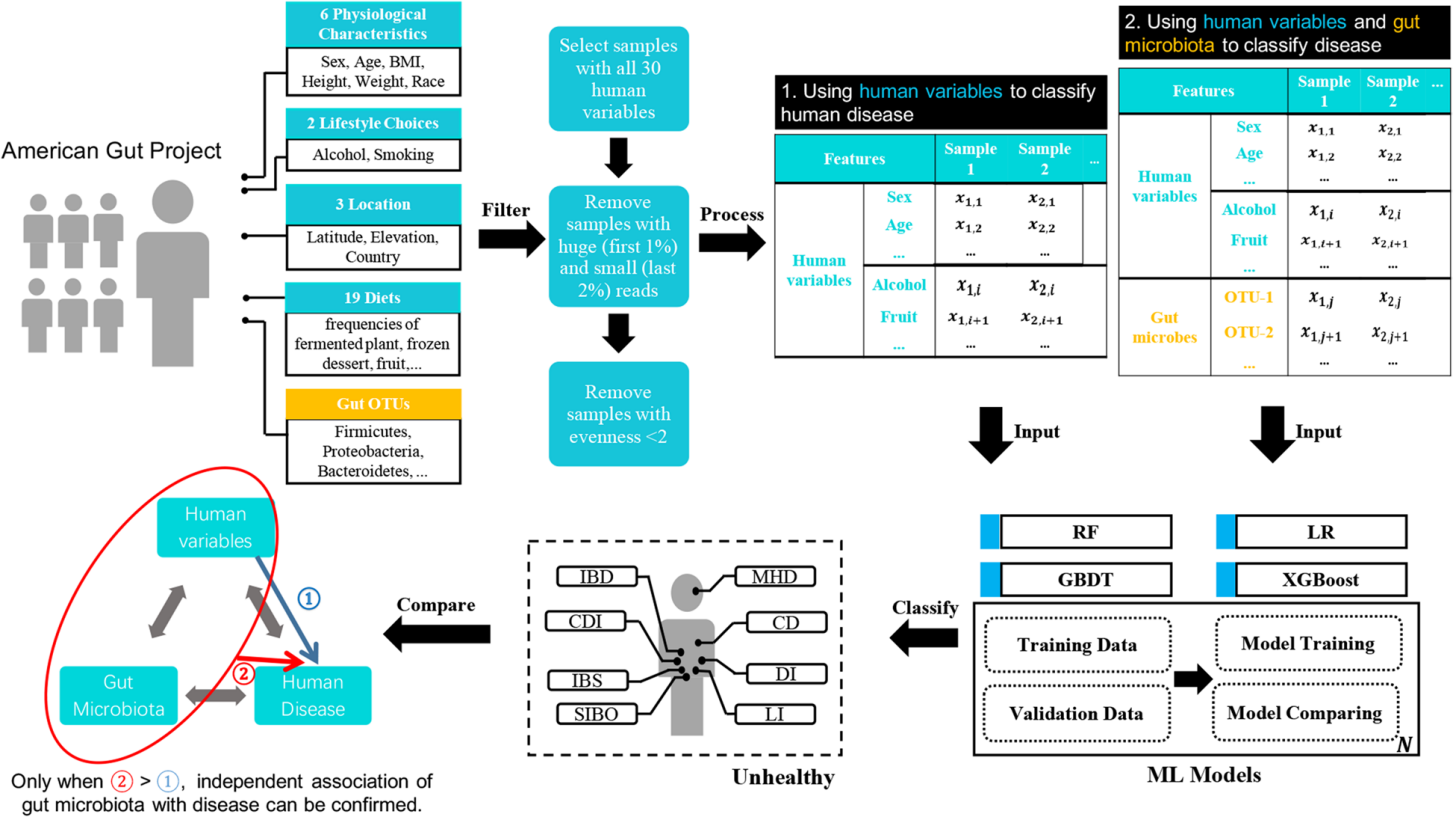
Workflow of disease classification models construction. We classified eight diseases (IBD: Inflammatory Bowel Disease; CDI: *C. difficile* Infection; IBS: Irritable Bowel Syndrome; SIBO: Small Intestinal Bacterial Overgrowth; DI: Diabetes; LI: Lactose Intolerance; CD: Cardiovascular Disease; MD: Mental Disorder) with 30 human variables (physiological characteristics, lifestyle, location, and diet) and gut microbial community data (OTUs) obtained from the American Gut Project database using four machine learning techniques (Random Forest, Gradient Boosting Decision Tree, Logistic Regression and eXtreme Gradient Boosting). We propose to build association models by including both human variables and gut microbiota, and assumed that when the performance of the model with both gut microbiota and human variables is better than the model with just human variables, the independent association of gut microbiota with the disease can be confirmed

Our approach is different from traditional association inference analysis, which tries to estimate the relationship between a single microbe and a disease. We focus on determining whether the whole gut microbiota is independently associated with human diseases by eliminating the influence of lifestyle factors using machine learning (ML) methods. The strength of association between gut microbiota and disease is evaluated by the classification performance of the ML models built with the microbiota. Although researchers have built large microbial datasets by merging different studies to explore the effect of gut microbiota on predicting diseases and mortality risks [18–20] via ML approaches, they neglected the impact of human lifestyle factors, resultant in the magnified predictive power of the microbiota. It is because human lifestyle factors influence both the gut microbiota and the disease progression. Besides, human lifestyle factors between enrolled healthy individuals (controls) and patients (cases) can be significantly different, and such differences could become the main contributor to the predictive power of the disease. It is necessary to build a well-performed disease classification model using both gut microbiota and human lifestyle and physiological variables in this condition. However, we argue that the independent gut microbiota-disease associations are real, only when the models’ classification performance with both gut microbiota and human variables is significantly better than the model built with just human lifestyle factors (Fig. 1). Conversely, when the models’ classification performance is inferior to the human-lifestyle-built model, either gut microbiota may not be associated with diseases, or a more suitable data enrollment criterion is needed.

Following the argument, we explored the classification power of the gut microbiota and human variables on multiple diseases using the AGP data with ML classification models. Key OTUs and human variables, consisting of lifestyle and dietary factors, were identified with high validity using multiple ML methods. The performance of OTUs and human variables was compared comprehensively to show the difference between their contributions to diseases. In addition, considering the widespread associations of gut microbes with multiple diseases, we use OTUs to judge the overall health status of humans, and individuals with at least one disease were classified as unhealthy. Although lots of associations with diseases were identified previously, our results showed that adding gut microbiota into human variables only enhanced the association strengths with IBD, irritable bowel syndrome, *C. difficile* infection, and unhealthy status. In addition, we reported the top 10 features (OTUs or human variables) used in the classification of these diseases, most of which were supported by previously published studies.

## Results

### Characteristics of the dataset

The dataset used in this study consisted of 7565 samples with 518 OTUs and 30 human variables (See Materials and methods for details). For human variables, there were 6 variables related to individuals’ physiological characteristics (age, sex, height, weight, body mass index (BMI), and race), 2 related to lifestyle choices (exercise and smoking frequencies), 3 related to location (latitude, elevation, and country), and 19 related to diet (frequencies of fruit, high-fat red meat, alcohol, and so on). For every sample, labels of eight diseases [cardiovascular disease (CD), small intestinal bacterial overgrowth (SIBO), mental disorders (MD), lactose intolerance (LI), diabetes (DI), (inflammatory bowel disease) IBD, irritable bowel syndrome (IBS), *C. difficile* infection (CDI) and Diabetes (DI)] that have been reported to be related to gut microbiota were extracted. Besides, a disease label named ‘unhealthy (UH)’ was added if a sample had at least one of eight diseases. The characteristics of the dataset, the demographic details of samples, and the number of male and female patients for each disease are shown in Tables S1 and S2.

For every disease, ML classification models were constructed using five types of features respectively: human variables only (Meta), OTU abundance only (OTUab), OTU occurrence only (OTUoc), both human variable data and OTU abundance (Meta-OTUab), and both human variable data and OTU occurrence (Meta-OTUoc). Models were trained and compared on identical training and validation data. For each type of feature, the best model was selected according to the AUC score. By comparing the performance of models with only Meta and models with both human variables and OTU information (Meta-OTUab and Meta-OTUoc), all diseases were classified into three categories: adding gut microbiota a) could improve, b) didn’t affect or c) reduced disease classification performance.

### Adding gut microbiota into human variables enhanced the association strengths with IBD

**Fig. 2:**
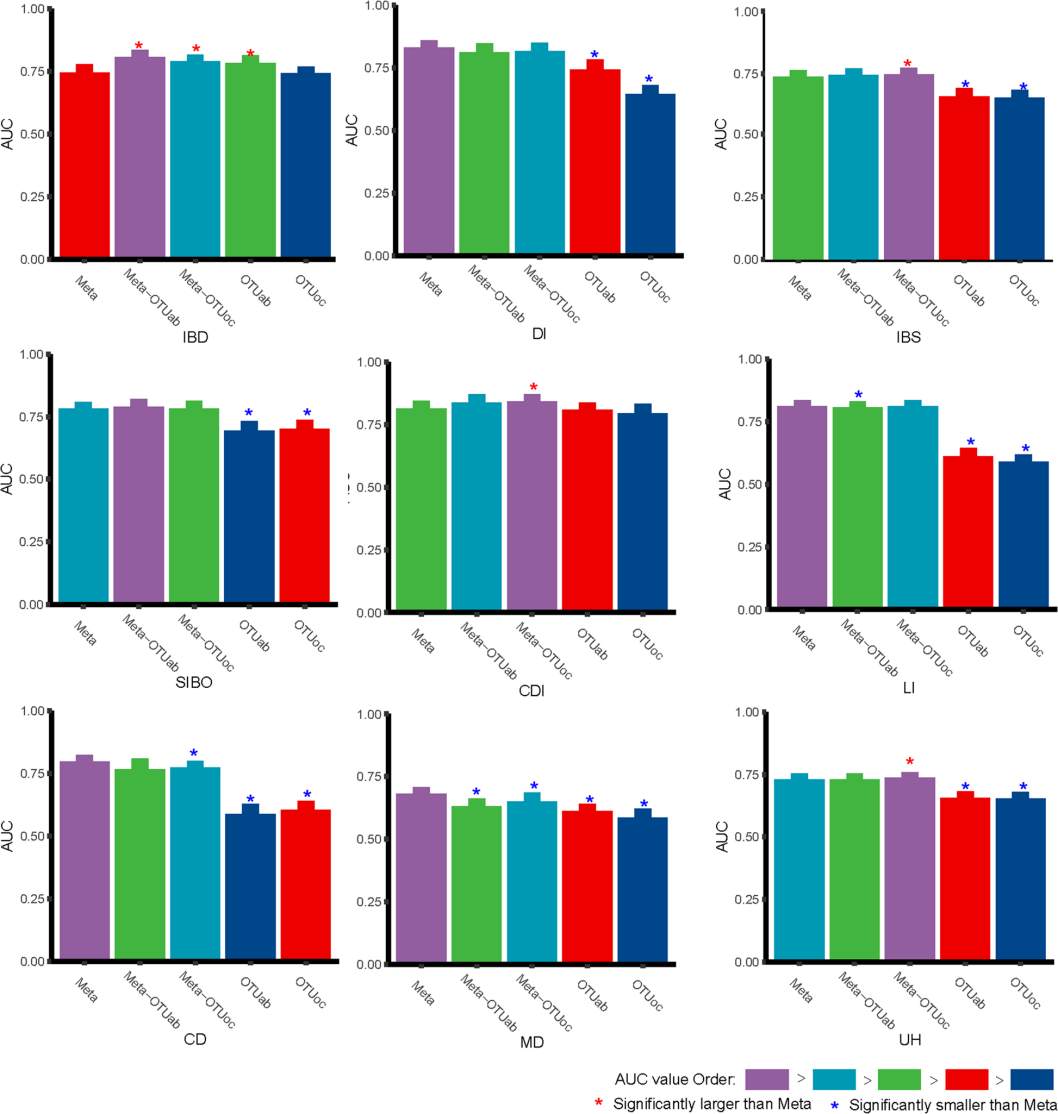
Comparing AUC values of nine diseases using five feature types

As a global public health concern, the incidence and prevalence of IBD, which is caused by gut dysbiosis, is increasing in developed and developing countries [21, 22]. In this study, 413 IBD patients from the AGP were included in the final dataset, and the results of the best models using five types of features are shown in Fig. 2 and Fig. S1. The model using human variable data only (Meta) as the feature achieved an AUC of 0.74677 ± 0.01240. Interestingly, the AUCs of models using OTUoc alone (0.74341 ± 0.00696) did not differ significantly from the models using Meta (*P* = 0.48579), and utilizing OTUab alone achieved significantly higher AUCs (0.78455 ± 0.00905; *P* = 0.00012), indicating that IBD classifier built with gut microbiota alone could be as good as with human variables. Additionally, the AUCs obtained using the combination of Meta and OTUs (Meta-OTUab: 0.80844 ± 0.00855 and Meta-OTUoc: 0.79028 ± 0.00637) were significantly higher than those obtained using Meta alone with *P* < 0.00001, suggesting that adding gut microbiota into human variables significantly enhanced the association strengths with IBD (Tables S3 and S4) and that the independent association of gut microbiota with IBD could be confirmed. It was noteworthy that the Meta-OTUoc achieved higher AUCs than those achieved with Meta-OTUab, which implied that, compared to the abundance of gut microbes, their occurrences are better features for the classification of IBD.

Next, we assessed the relative roles of Meta or OTUs in the best ML model for classifying IBD with Meta, Meta-OTUab, and Meta-OTUoc. We ranked the features according to their weights and calculated the average rank after repeating the model training process ten times. As shown in Table 1, we found that the top 10 features for the three types of features were distinct. For the model using human variable data only (Meta) as features, the top 10 most important human variables for classifying IBD comprised six dietary characteristics (the frequencies of vitamin B, probiotics, salted snacks, milk cheese, frozen dessert, and vitamin D), two basic physical characteristics (BMI and age) and two geographical location features (elevation and latitude). For the model using Meta-OTUab as features, except for three dietary characteristics (the frequencies of probiotics, vitamin D, and vitamin B), the other seven of the top 10 features for classifying IBD were all OTUs (four *Clostridiales*, one *Bacteroidales*, one *Erysipelotrichales*, and one *Enterobacteriales*). When using Meta-OTUoc as features to classify IBD, the results changed in that, except for four human variables (probiotics, exercise, weight, and Caucasian), the other six of the top 10 features were all OTUs of *Clostridiales*.

**Table 1:** Top 10 most important features using three types of feature sets for IBD

Meta		Meta-OTUab		Meta-OTUoc	
Feature Name	Rank	Feature Name	Rank	Feature Name	Rank
ELEVATION	5	PROBIOTIC_FREQUENCY	3.7	p_Firmicutes;c_Clostridia;o_Clostridiales;f_Lachnospiraceae;g_;s_	5
VITAMIN_B_SUPPLEMENT_FREQUENCY	6	p_Firmicutes;c_Erysipelotrichi;o_Erysipelotrichales;f_Erysipelotrichaceae;g_Holdemania;s_	4.7	p_Firmicutes;c_Clostridia;o_Clostridiales;f_Ruminococcaceae;g_Ruminococcus;s_	14
PROBIOTIC_FREQUENCY	6.3	p_Firmicutes;c_Clostridia;o_Clostridiales;f_Lachnospiraceae;g_;s_	9.8	PROBIOTIC_FREQUENCY	15.6
LATITUDE	7.3	p_Bacteroidetes;c_Bacteroidia;o_Bacteroidales;f_Rikenellaceae;g_Alistipes;s_indistinctus	13.4	EXERCISE_FREQUENCY	24.7
SALTED_SNACKS_FREQUENCY	7.7	p_Firmicutes;c_Clostridia;o_Clostridiales;f_Lachnospiraceae;g_Coprococcus;s_	14.5	p_Firmicutes;c_Clostridia;o_Clostridiales;f_Ruminococcaceae;g_;s_	25.3
AGE_CORRECTED	7.7	p_Firmicutes;c_Clostridia;o_Clostridiales;f_Ruminococcaceae;g_Ruminococcus;s_	21	p_Firmicutes;c_Clostridia;o_Clostridiales;f_;g_;s_	45.4
BMI	7.9	p_Proteobacteria;c_Gammaproteobacteria;o_Enterobacteriales;f_Enterobacteriaceae;g_Morganella;s_	22.3	WEIGHT_KG	48.5
MILK_CHEESE_FREQUENCY	8.1	VITAMIN_D_SUPPLEMENT_FREQUENCY	23.3	p_Firmicutes;c_Clostridia;o_Clostridiales;f_Lachnospiraceae;g_;s_	50.7
FROZEN_DESSERT_FREQUENCY	9.7	p_Firmicutes;c_Clostridia;o_Clostridiales;f_Lachnospiraceae;g_[Ruminococcus];s_	29.1	Caucasian	51.2
VITAMIN_D_SUPPLEMENT_FREQUENCY	9.7	VITAMIN_B_SUPPLEMENT_FREQUENCY	31.3	p_Firmicutes;c_Clostridia;o_Clostridiales;f_Lachnospiraceae;g_;s_	51.3

### Adding gut microbiota occurrence information improved the association strength with IBS, CDI, and unhealthy status

Irritable bowel syndrome (IBS) and *C. difficile* infection (CDI) are widely reported to be closely related to gut microbes and some diet habits [3, 23]; therefore, we hypothesized that adding microbes into human variables will improve the classification of these two conditions. To investigate the potential use of the out composition to classify the health of individuals, we defined a sample as unhealthy if it was obtained from an individual with any one of the eight diseases. Finally, we obtained 2921 unhealthy samples (UH) containing at least one of those eight diseases for training the models using OTUs or Meta alone and in combination. As shown in Fig. 2, comparing to Meta, Meta-OTUoc provided the significantly higher AUCs (IBS: 0.73953 ± 0.00580, CDI: 0.84252 ± 0.00959 and UH: 0.73582 ± 0.00280) in classifying all these three diseases with *P* = 0.00107, 0.00003, and 0.00001. And, the AUCs obtained using Meta-OTUab were not significantly different from those obtained using Meta for all these three diseases, indicating that adding gut microbiota occurrence information improved the association strength with IBS, CDI, and unhealthy status. Surprisingly, for CDI, the AUC obtained using OTUab (0.80916 ± 0.00922) and OTUoc (0.79603 ± 0.01700) both did not differ significantly from that obtained using Meta (*P* = 0.24377 and 0.02344), indicating that CDI can be classified accurately based on the abundance information of gut microbes alone. However, for IBS and UH, the AUC obtained using OTUab and OTUoc were all significantly lower than that obtained using Meta.

When calculating the weight for each feature in the models with Meta-OTUoc, we identified the top 10 most important features for classifying these three diseases (Table 2). When using Meta-OTUoc as the features to classify IBS, except for one OTU annotated to *Clostridia*, the other nine of the top 10 most important features were all human variables, including three dietary characteristics (the frequencies of milk cheese, probiotics, and milk substitute), four basic physical characteristics (age, sex, weight, and height), one geographical location feature (latitude) and one lifestyle (exercise frequency). For the CDI, the top 10 most important human variables comprised two dietary characteristics (the frequencies of vitamin B and probiotics), two basic physical characteristics (BMI and weight), one lifestyle (exercise frequency), and five OTUs (one *Erysipelotrichales*, three *Clostridiales*, and one *Bacteroidales*). When classifying UH, except for three OTUs annotated to *Clostridia*, the other seven of the top 10 OTUs were all human variables, including five dietary characteristics (the frequencies of milk cheese, probiotics, milk substitute, frozen dessert, and vitamin B), one basic physical characteristic (age) and one lifestyle (poultry frequency).

**Table 2:** Top 10 features using Meta-OTUoc for classifying IBS, CDI, and UH

	IBS	CDI	UH
1	LATITUDE	p_Firmicutes;c_Erysipelotrichi;o_Erysipelotrichales;f_Erysipelotrichaceae;g_;s_	MILK_CHEESE_FREQUENCY
2	MILK_CHEESE_FREQUENCY	p_Firmicutes;c_Clostridia;o_Clostridiales;f_Lachnospiraceae;g_;s_	PROBIOTIC_FREQUENCY
3	PROBIOTIC_FREQUENCY	VITAMIN_B_SUPPLEMENT_FREQUENCY	MILK_SUBSTITUTE_FREQUENCY
4	AGE_CORRECTED	BMI	p_Firmicutes;c_Clostridia;o_Clostridiales;f_;g_;s_
5	female	WEIGHT_KG	p_Firmicutes;c_Clostridia;o_Clostridiales;f_Lachnospiraceae;g_;s_
6	WEIGHT_KG	p_Firmicutes;c_Clostridia;o_Clostridiales;f_Ruminococcaceae;g_;s_	AGE_CORRECTED
7	MILK_SUBSTITUTE_FREQUENCY	EXERCISE_FREQUENCY	FROZEN_DESSERT_FREQUENCY
8	HEIGHT_CM	p_Firmicutes;c_Clostridia;o_Clostridiales;f_Ruminococcaceae;g_Oscillospira;s_	POULTRY_FREQUENCY
9	EXERCISE_FREQUENCY	PROBIOTIC_FREQUENCY	VITAMIN_B_SUPPLEMENT_FREQUENCY
10	p_Firmicutes;c_Clostridia;o_Clostridiales;f_;g_;s_	p_Bacteroidetes;c_Bacteroidia;o_Bacteroidales;f_[Barnesiellaceae];g_;s_	p_Firmicutes;c_Clostridia;o_Clostridiales;f_;g_;s_

### Adding gut microbiota showed no effect on association strengths with DI, SIBO, LI, and CD

Recently, gut microbes were also reported to be related to Diabetes (DI), small intestinal bacterial overgrowth (SIBO), lactose intolerance (LI), cardiovascular disease (CD), and mental disorders (MD) [4–7, 24]. As shown in Fig. 2, the AUCs of using gut microbiota alone (OTUab and OTUoc) were both significantly lower than that obtained using Meta for all these five diseases, indicating that gut microbiota alone is not a good classifier of IBD as human variables. However, it is noteworthy that the AUCs obtained using the combination of Meta and OTUs (Meta-OTUab and Meta-OTUoc) did not differ significantly from those obtained using Meta alone (Meta) for DI and SIBO, suggesting that adding gut microbiota into human variables showed no effect on association strengths with DI and SIBO. Besides, the AUCs obtained using Meta-OTUoc did not differ significantly from those obtained using Meta alone (Meta) for LI, suggesting that adding gut microbiota occurrence features showed no effect on the association strength with IBS. And, the AUCs obtained using Meta-OTUab did not differ significantly from those obtained using Meta alone (Meta) for CD, suggesting that adding gut microbiota abundance features showed no effect on the association strength with CD. In addition, neither Meta nor OTUs nor their combination provide good performance for classifying MD.

**Fig. 3:**
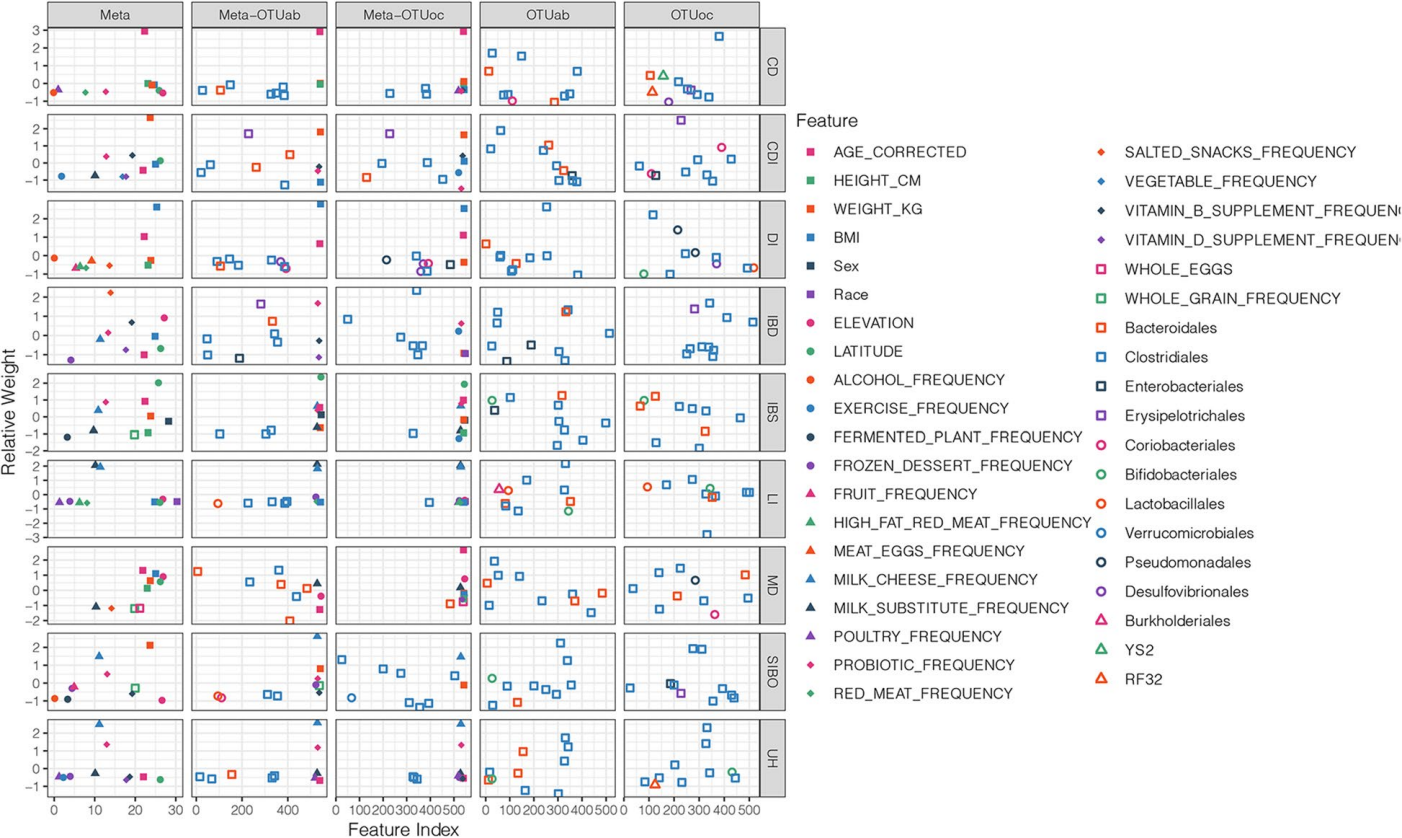
Feature distribution for the best model with the highest AUC. Different features are marked with various colors and shapes. OTUs are annotated at the order level. In all subgraphs, the orders of host variables and OTUs are fixed and unified, and OTUs are sorted according to their average sizes reversely

The top 10 most important features for classifying DI, SIBO, LI, and CD with the combination of Meta and OTUs archiving the highest AUCs were identified according to the feature weights (Fig. 3 and Table 3). The top 10 most important features for classifying DI with Meta-OTUoc comprised seven OTUs (two *Clostridiales*, two *Desulfovibrionales*, one *Coriobacteriales*, one *Enterobacteriales*, and one *Pseudomonadales*) and three basic physical characteristics (BMI, age, and weight). When classifying SIBO with Meta-OTUab, except for five dietary characteristics (the frequencies of milk cheese, whole grain, frozen dessert, and vitamin B) and one basic physical characteristic (weight), four of the top 10 most important features were all OTUs (two *Clostridiales*, one *Coriobacteriales* and one *Lactobacillales*). The top 10 most important variables for classifying LI comprised five dietary characteristics (the frequencies of milk substitute, milk cheese, frozen dessert, high-fat red meat, and red meat), one lifestyle (poultry frequency), two basic physical characteristics (BMI and Caucasian), and one OUT annotated to *Clostridiales*. It is reasonable that the two most important human variables for classifying LI are the frequencies of milk substitute and milk cheese, followed by one race-related feature (Caucasian). It is noteworthy that CD was classified mainly by seven OTUs, six of which were annotated to *Clostridiales*, and three basic physical characteristics (age, weight, and height). Interestingly, BMI or weight was of the most 10 most important features for all four diseases.

**Table 3:** Top 10 features using a combination of Meta and OTUs for classifying DI, SIBO, LI, and CD

	DI (Meta-OTUoc)	SIBO (Meta-OTUab)	LI (Meta-OTUoc)	CD (Meta-OTUab)
1	BMI	MILK_CHEESE_FREQUENCY	MILK_SUBSTITUTE_FREQUENCY	AGE_CORRECTED
2	AGE_CORRECTED	PROBIOTIC_FREQUENCY	MILK_CHEESE_FREQUENCY	WEIGHT_KG
3	p_Firmicutes;c_Clostridia;o_Clostridiales;f_Lachnospiraceae;g_;s_	WHOLE_GRAIN_FREQUENCY	FROZEN_DESSERT_FREQUENCY	p_Firmicutes;c_Clostridia;o_Clostridiales;f_Ruminococcaceae;g_;s_
4	WEIGHT_KG	FROZEN_DESSERT_FREQUENCY	POULTRY_FREQUENCY	p_Bacteroidetes;c_Bacteroidia;o_Bacteroidales;f_[Barnesiellaceae];g_;s_
5	p_Proteobacteria;c_Deltaproteobacteria;o_Desulfovibrionales;f_Desulfovibrionaceae;g_Desulfovibrio;s_	WEIGHT_KG	Caucasian	p_Firmicutes;c_Clostridia;o_Clostridiales;f_Lachnospiraceae;g_Coprococcus;s_
6	p_Actinobacteria;c_Coriobacteriia;o_Coriobacteriales;f_Coriobacteriaceae;g_;s_	p_Firmicutes;c_Clostridia;o_Clostridiales;f_Lachnospiraceae;g_[Ruminococcus];s_	ELEVATION	HEIGHT_CM
7	p_Proteobacteria;c_Deltaproteobacteria;o_Desulfovibrionales;f_Desulfovibrionaceae;g_Bilophila;s_	p_Actinobacteria;c_Coriobacteriia;o_Coriobacteriales;f_Coriobacteriaceae;g_Collinsella;s_aerofaciens	HIGH_FAT_RED_MEAT_FREQUENCY	p_Firmicutes;c_Clostridia;o_Clostridiales;f_Ruminococcaceae;g_Ruminococcus;s_
8	p_Proteobacteria;c_Gammaproteobacteria;o_Enterobacteriales;f_Enterobacteriaceae;g_;s_	p_Firmicutes;c_Clostridia;o_Clostridiales;f_Ruminococcaceae;g_Oscillospira;s_	p_Firmicutes;c_Clostridia;o_Clostridiales;f_Lachnospiraceae;g_;s_	p_Firmicutes;c_Clostridia;o_Clostridiales;f_Ruminococcaceae;g_;s_
9	p_Firmicutes;c_Clostridia;o_Clostridiales;f_Ruminococcaceae;g_;s_	p_Firmicutes;c_Bacilli;o_Lactobacillales;f_Streptococcaceae;g_Streptococcus;s_	BMI	p_Firmicutes;c_Clostridia;o_Clostridiales;f_Lachnospiraceae;g_;s_
10	p_Proteobacteria;c_Gammaproteobacteria;o_Pseudomonadales;f_Pseudomonadaceae;g_;s_	VITAMIN_B_SUPPLEMENT_FREQUENCY	RED_MEAT_FREQUENCY	p_Firmicutes;c_Clostridia;o_Clostridiales;f_;g_;s_

## Discussion

### Important OTUs for human diseases

For IBD, adding gut microbiota to human variables can achieve better results than that achieved using human variables alone and Meta-OTUab achieved the highest AUC. Among the top 10 most important features used to classify IBD with Meta-OTUab, eight were OTUs with four belonged to the *Clostridiales* order. At the family level, two were annotated to *Ruminococcaceae*, which have been reported as a prominent family in IBD, especially two species, *Ruminococcus torques*, and *Ruminococcus gnavus* [25], and two were annotated to *Lachnospiraceae*, which is also reported to be related to IBD [26]. The physiologic niche of *Ruminococcus gnavus* was speculated to be mucolytic, with dramatic changes in this species affecting the delicate equilibrium of the mucus layer and potentially increasing the intestinal permeability in IBD patients [25]. One belonged to the *Klebsiella* genus. *Klebsiella* is an intestinal pathobiont that can produce a cytotoxin (tillivaline) and is thought to be involved in the pathogenesis of IBD [27]. For the unhealthy status classification, three of the top 10 features with the highest weight belonged to the *Clostridiales* order. Under the *Clostridiales* order, the *Lachnospiraceae* family has been associated with many human diseases, such as IBD [26], irritable bowel syndrome [28], type 1 diabetes [29, 30], *Clostridium difficile* infection [31] and liver cirrhosis [32]. The *Ruminococcaceae* family was also reported to be related to *Clostridium difficile* infection [31] and type 1 diabetes [29]. The other two most important OTUs for unhealthy status classification belonged to the *Bacteroides* genus and the *Bifidobacterium* genus. According to the previous research, *Bacteroides* genus was associated with several human diseases, including five gut diseases (irritable bowel syndrome [28], *Clostridium difficile* infection [31], colorectal carcinoma [33], Crohn’s disease [34, 35] and infectious colitis [36]), type 1 diabetes [29, 30] and liver cirrhosis [37]. From the OTUs distribution across disease states (Fig. 3), most of the important OTUs are from the order ‘Clostridiales’ and many relatively rare OTUs (Feature Indexes range from 300 to 400) play important roles in disease predictions. By comparing the distributions of features of ‘OTUoc’ and ‘Meta-OTUoc’, it is found that effects of abundant OTUs, which are important in ‘OTUoc’, weaken in ‘Meta-OTUoc’, which may be the reason that there are more potential associations between abundant OTUs and host variables than the rare OTUs. For IBD, by comparing distributions of ‘Meta’ and ‘Meta-OTUab’ which achieves the highest AUCs, we found that predictive effects of OTUs are stronger than most of the host variables except for several diet factors, which proves the value of microbiota to IBD classification again. For SIBO, a small intestinal disease, adding gut microbiota from feces into human variables showed no effect on association strength, which means that feces may be not the correct material to investigate for SIBO.

### Important human variables for human diseases

Human variables showed a strong efficiency in human disease classification. According to our results shown in Fig. 3, the basic physiological characteristics (BMI, age, height, and weight) are the most important human variables correlated to most diseases, followed by location factors (latitude and elevation) and the frequency of probiotics, milk cheese, and alcohol intake. BMI and age were found to be important classifiers of all seven diseases except lactose intolerance, which is supported by a cross-sectional study of pre-specified demographic and clinical data [38]. Multiple pieces of evidence from experimental and observational studies showed that for a substantial proportion of patients with IBS, their symptoms were associated with the ingestion of specific foods, such as milk, which contain lactose, a disaccharide that is not effectively digested by many adults worldwide [39, 40]. Additionally, evidence exists to suggest that probiotics may exert an effect on IBS through various mechanisms [41]. Following previous studies, we found that milk cheese and probiotics intake were two of the most important types of human variables in addition to latitude and age for classifying IBS (Table 2). For DI classification, we found that BMI and age were the two most important types of human variables, which was supported by a cross-sectional study [38]. We found that the most important health feature for classifying SIBO was the frequency of probiotics intake, which was supported by a previous systematic review [42]. The review and meta-analysis showed that probiotics are both safe and effective for preventing SIBO. We also found that the frequency of cheese and milk intake are two of the most important features for LI classification. This finding is not surprising because the breakdown of nondigested lactose causes LI; therefore, LI management usually involves excluding milk and milk products from the diet. It is noteworthy that the race-related feature was also among the top 10 health features for LI classification. This discovery was validated by the previous reports that lactase persistence varies among different human populations [43]. Age and sex were found to be the two most important features for classifying CD. This is a reasonable conclusion because age has been reported as one of the most powerful risk factors for developing CD [44] and there is a higher prevalence of CD in men than in women [45].

### Removing probiotics, vitamin B, and vitamin D

The frequency of probiotics intake was among the top 10 features of IBD, IBS, CDI, SIBO, and UH; the frequency of eating vitamin B was among the top ten features of IBD, CDI, SIBO, and UH; the frequency of vitamin D intake was among the top 10 features of IBD and MD. Some samples may have been obtained from individuals who adopted these three dietary habits advised by the clinician; therefore, we repeated our analysis after removing these three health features. As the results are shown in Fig. S2 and Table S5, we found that, after removing the frequency of probiotics and vitamins B and D intake, Meta-OTUab, Meta-OTUoc, OTUab, and OTUoc all showed significantly better results than Meta for classifying IBD; Meta-OTUab, Meta-OTUoc and OTUab all showed significantly better results than Meta for classifying CDI; Meta-OTUoc showed significantly better results than Meta for classifying SIBO and UH; Meta-OTUab and Meta-OTUoc showed no significantly different with Meta for classifying DI, IBS, and LI. While, Meta-OTUab, Meta-OTUoc, OTUab, and OTUoc all showed significantly worse results than Meta for classifying CD and MD. These results mean adding gut microbiota into human variables enhanced the association strengths with IBD, SIBO, CDI, and UH, suggesting that the independent associations do exist between gut microbiota and IBD, SIBO, CDI, and UH. Besides, adding gut microbiota showed no effect on association strengths with DI, IBS, and LI. The top 10 most important features for disease classification after removing the frequency of probiotics and vitamins B and D intake are shown in Table S6.

### The best model for different diseases and performance changed with OTU numbers

**Fig. 4:**
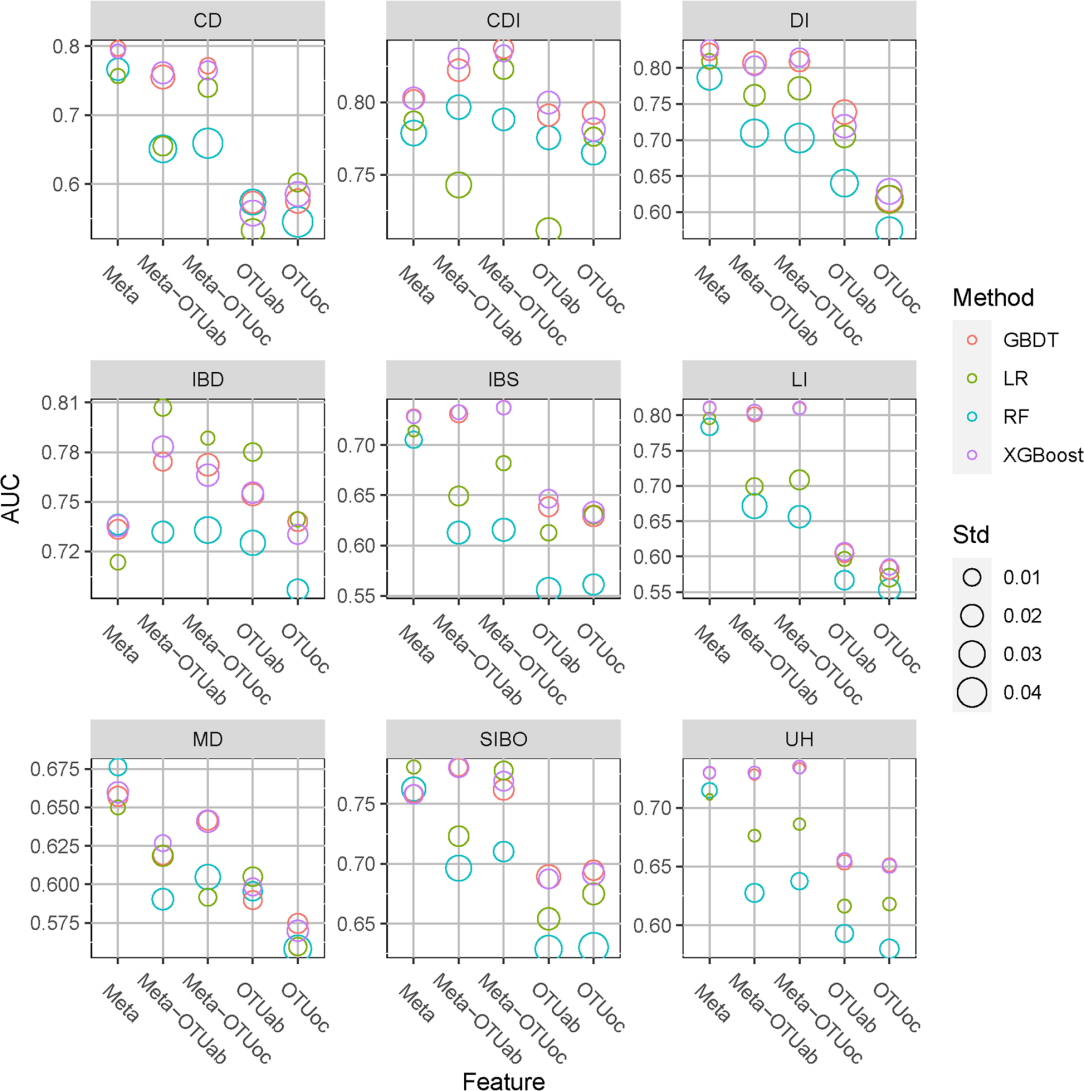
Performances of four machine learning methods in different characteristics and disease prediction. The color of the open circle represents different machine learning methods, and the size represents the standard deviation

We validated the performances of four ML methods in different features and disease prediction by showing AUCs of four machine learning methods on the validation dataset for five feature types and nine diseases for comparison (Fig. 4). We found that different ML methods achieved the best performances for different features and different diseases, with the XGBoost and GBDT methods performing similar and AUCs of these two methods better than other methods in most disease classification tasks except the IBD and MD. However, LR models generated the highest AUCs for IBD prediction using the other four types of features except for using Meta and for SIBO prediction using Meta and Meta-OTUoc. RF model obtained the highest AUC for MD prediction using Meta. These results suggested that we can combine the advantages of the four machine learning methods to improve the overall prediction effect.

**Fig. 5:**
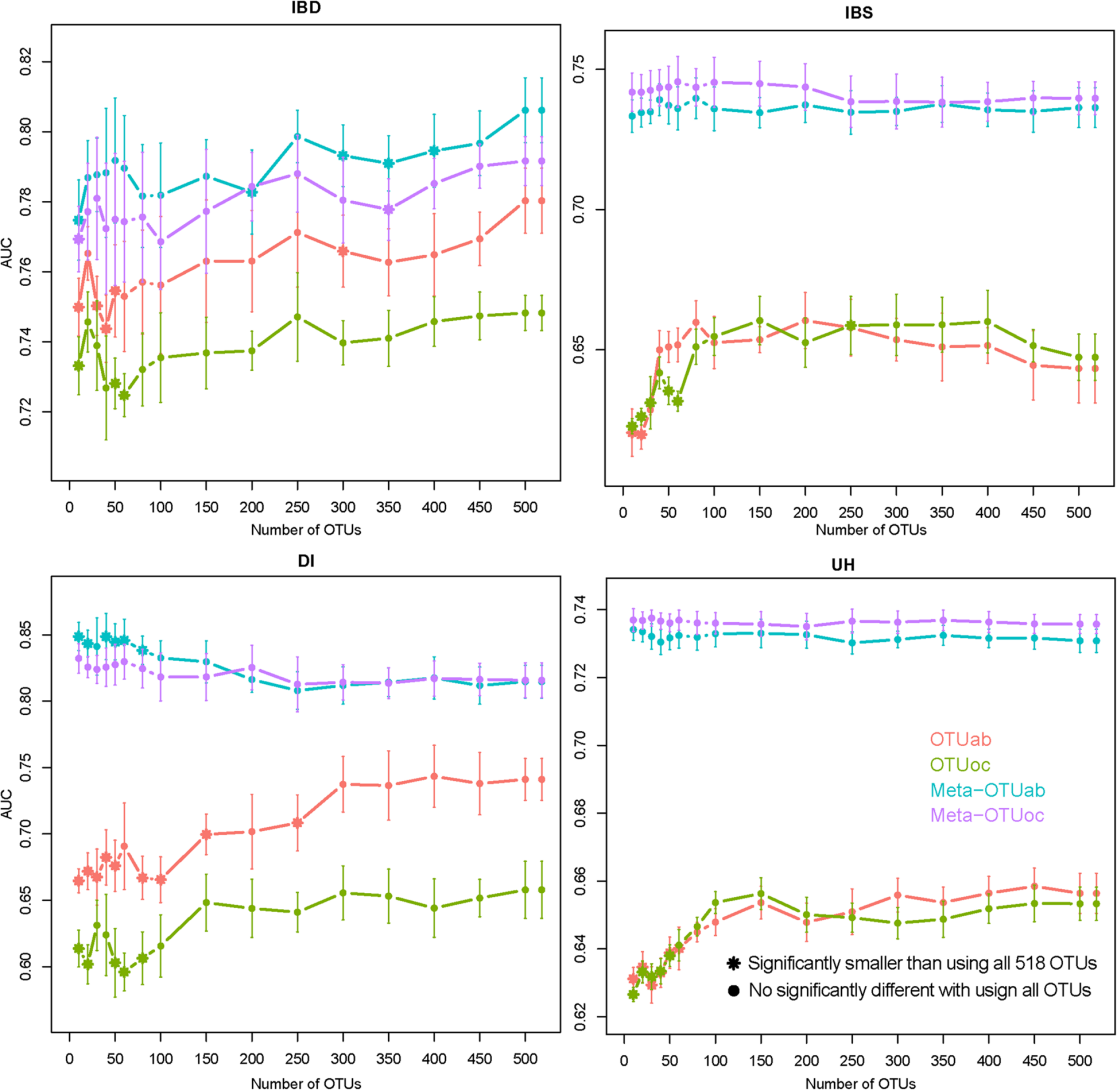
Changes in the AUC of the optimal model with the number of OTUs. The optimal model for four diseases (IBD: Inflammatory Bowel Disease; IBS: Irritable Bowel Syndrome; DI: Diabetes; UH: Unhealthy status)

When there are too many OTUs as input features, the models may be overfitted. Spearman’s correlations of the human variables indicated modest, or high, inter-correlations between some factors (Table S7). Therefore, we evaluated the changes in classification performance for the four diseases (IBD, IBS, DI, and UH) in the optimal model result with the number of OTUs. The changes in AUCs obtained using a reduced number of OTUs are shown in Fig. 5. We found that using only some of the OTUs achieved better or equal results than using all 518 OTUs. Especially for IBD, using only the top 3% of OTUs (20 OTUs) achieved no significantly different results with using all OTUs for all four types of input features. For IBS, the OTUs number showed no significant effect on the combination of OTUs and Meta, but the top 5.8% of OTUs (30 OTUs) and 7.7% (40 OTUs) for OTUab and OTUoc respectively generated no significantly different results with using all OTUs. Interestingly, for DI, the top 2% of OTUs (10 OTUs) for Meta-OTUab generated significantly better results than using all OTUs combing Meta. The OTU set with the best classification results was different for the four diseases. These OTU sets can be used as biomarkers for the corresponding diseases.

### Influence of adding gut microbiota diversity feature on models’ performance

In our experiments, abundance or occurrence of OTUs is used in modeling directly, instead of features that summarize the structure of microbial community (E.g. alpha diversity). Loss of diversity is reported to be associated with many diseases such as IBD, obesity, and diabetes [46, 47]. We also try adding the value of alpha diversity (Shannon Index and Simpson Index) as new features and AUCs of re-trained models are shown in Fig. S3 and Table S8. Compared with results without microbial diversity features (Fig. 2), these two features don’t improve the prediction performance of the machine learning model. For models with feature type ‘OTUab’ or ‘Meta-OTUab’, it may be the reason that richness and evenness of microbiota have already been implied in OTUs’ abundance information fully. For models with ‘OTUoc’ or ‘Meta-OTUoc’, one possible reason is that small OTUs have a greater impact on prediction performance as shown in Fig. 2, and relative abundance of small OTUs can be noisy and biased, due to the influence of sequences amplification and compositional bias, which limits the utility of abundance information in modeling.

Similarly, one notable result is that, as shown in Fig. 2, when we used only gut microbes for disease classification, the OTUab achieved better results than that achieved using OTUoc, which can be explained by the greater loss of information using OTUoc than when using OTUab. However, after combination with human variables, the performance of Meta-OTUoc surpassed the Meta-OTUab in most cases. This difference might be caused by two reasons. One is that, for disease classification, the information provided by human variables and microbial abundance were overlapped. The other is that OTUs abundance information is less robust than the occurrence which is less affected by the sequencing process.

## Conclusions

We evaluated the feasibility of gut microbiota on multiple diseases prediction and compared its predictive validity with human variables comprehensively. Our results showed that gut microbiota has distinct performances in classifying different diseases. Combining human variables and gut microbiota achieved the best performances in predicting IBD, IBS, CDI, and unhealthy status, indicating independent associations between gut microbiota and these diseases. OTU-based prediction results were similar to Meta-based prediction results in predicting IBD and CDI, so we can predict these diseases by just measuring the gut microbe community. Although gut microbiota is also reported to be associated with LI, CD, and MD, they do not predict these diseases well. Further investigations about associations between gut microbial community and diseases are still necessary, except IBD and unhealthy status, and whether gut microbes can be used as biomarkers for other diseases still needs to be explored. We have reported the top 10 features (microbes or human variables) of these diseases and most were supported by previously published reports. More researches on these features may improve our understanding of the molecular mechanism of human diseases.

## Materials and methods

### Data sources

We downloaded the OTU table (11-packaged/fecal/100 nt/ag_fecal.biom) and human variables (11-packaged/fecal/100 nt/ag_fecal.txt) from the latest version (updated in January 2018) of the AGP database available at ftp://ftp.microbio.me/AumericanGut/latest. The original OTU table was saved as a binary file (.biom), which was converted manually to plain text with Python Script, which is available at GitHub (https://github.com/tinglab/kLDM.git). The original gut microbial abundance table (OTU table) contained 15,158 samples and 24,114 OTUs selected by applying a 97% similarity cutoff with SortMeRNA [48] defined by the AGP consortium. The OTUs were mapped to the Greengenes Database [49] to identify their taxonomy. Each cell in the OTU table presents the abundance of its corresponding OTUs in a specific sample. The original human variables file (Meta table) contained 15,158 samples and 523 factors related to physicochemical parameters of fecal samples, dietary habits, lifestyle choices, and some diseases. Each cell in the Meta table presents the measured value of its corresponding meta-data in a specific sample. As the data used is publicly available, ethical approval was not required.

### Data preprocessing

We selected 30 items of human variables to classify disease, including individuals’ physiological characteristics, lifestyle, location, and diet. Among these 30 items, six were related to physiological characteristics; two were associated with lifestyle choices; three were associated with location; the remaining 19 related to diet (frequencies of fermented plant, frozen dessert, fruit, high-fat red meat, home-cooked meals, alcohol, red meat, meat eggs, milk substitute, milk cheese, olive oil, probiotics, salted snacks, seafood, vegetable, vitamin D supplement, vitamin B supplement, whole grain, and whole eggs). The values of frequency-related human variables were categorized as follows: ‘Never’, ‘Rarely’ (less than once/week), ‘Occasionally’ (1–2 times/week), ‘Regularly’ (3–5 times/week), and ‘Daily’. For convenience, these categories were recoded as integers from 1 to 5 (where 1 represents ‘never’ and 5 represents ‘daily’) according to their frequencies (Table S1). The values of human variables were missing for some samples; therefore, the samples with complete sets of 30 human variable data items were selected for the following analysis. Samples with huge (first 1%) and small (last 2%) reads, as well as those with evenness < 2 were removed. For all selected samples, OTUs were selected from the top 50% based on their average abundance in nonzero samples. An additional filtration step was then performed to remove the rarefied microbes that appeared in less than 20% of the total samples. We used the relative abundance of OTUs and performed log conversion to reduce the data range, followed by normalization. Finally, 7565 samples with 518 OTUs and 30 human variables were retained for further analysis.

Eight diseases (CD, SIBO, MD, LI, DI, IBD, IBS, CDI, and DI) that have been reported in previous studies to be related to gut microbiome were selected. The samples from individuals affected by any of these eight diseases were treated as unhealthy (UH). Information about individuals’ disease status was extracted, and the samples from individuals with diseases were labeled. The characteristics of the dataset, the demographic details of samples, and the number of male and female patients for each disease are shown in Table S2.

### Machine learning models training and evaluation

To evaluate the confound association between disease with gut microbiota and human variables, four machine learning (ML) techniques (RF, Random Forest; GBDT, Gradient Boosting Decision Tree; LR, Logistic Regression; XGBoost, eXtreme Gradient Boosting) were used to build the model, and the AUC scores were calculated to compare their performance (Fig. 1). All models were implemented using Python 2.7 and *scikit-*learn (version 0.16.1) and xgboost (version 0.82) libraries. For every disease, four types of ML models were trained with five-fold cross-validation using training data, including 80% of all samples, and the model providing the best performance was selected based on the maximal AUC. Considering that the positive samples labeled with diseases occupied a tiny proportion, an equal number of negative samples were randomly selected for model training. The optimal model was then evaluated and compared using the validation dataset comprising the remaining 20% of samples.

In addition to the different model types, five combinations of features were used to construct the following separate models to capture the best features for classifying each disease: human variables only (Meta), OTU abundance only (OTUab), OTU occurrence only (OTUoc), both human variable data and OTU abundance (Meta-OTUab), and both human variable data and OTU occurrence (Meta-OTUoc). The OTU occurrence was determined based on the existence of OTUs only. Models using different combinations of features were trained and compared using an identical dataset. For each type of feature, the best model with the maximal AUC score was selected from the four types of models.

To assess the significance of differences in the model performance among the five types of features, the model training process was repeated 10 times with a random selection of training data. The AUC scores were presented as the mean ± standard deviation. For each disease, paired sample *t*-tests were used to compare the differences in AUC values between the feature type ‘Meta’ and the four other feature types (‘OTUab’, ‘OTUoc’, ‘Meta-OTUab’ and ‘Meta-OTUoc’). In the statistical analysis, Bonferroni correction was used to adjust for the multiple testing error. In considering nine diseases and four comparisons, we tested 36 independent hypotheses using the same data at the 0.05 significance level, and instead of using a *P*-value threshold of 0.05, we use a stricter threshold of 0.0014.

### Identification of microbial biomarkers of diseases

For the best-performing model of each disease, the weights of each feature were calculated. The top ten features (OTUs or Meta) with the highest absolute weights were selected as the biomarkers for the disease. We then obtained the taxa of these OTUs and verified their relationships to the disease by searching published literature or databases. In this study, we used the Human Microbe-Disease Association Database (HMDAD) (http://www.cuilab.cn/hmdad), which is a curated collection of microbe-disease associations from previous microbiota studies. The OTUs with high weights that were verified were treated as microbial biomarkers of the disease.

## Supplementary Information

**Additional file 1: Fig. S1.** ROC curves for a selection of the best classification models of eight diseases. **Fig. S2.** Comparing the differences of AUCs of nine diseases using five feature types after removing probiotics, vitamin B, and vitamin D. **Fig. S3.** Comparing the differences of AUCs of nine diseases using five feature types after adding gut microbial diversity. **Table S1.** Frequency of diet and lifestyle factors. **Table S2.** Basic information of the dataset. **Table S3.** Comparing AUC values of nine diseases using five feature types. **Table S4.** AUCs, sensitivity, and specificity for five types of features of the best model selected according to the AUC score. **Table S5.** Comparing AUC values of nine diseases using five feature types after removing probiotics, vitamin B, and vitamin D. **Table S6.** Top 10 features after removing probiotics, vitamin B, and vitamin D for all diseases. **Table S7.** Spearman’s correlations of the human variables and disease. **Table S8.** Comparing AUC values of nine diseases using five feature types with adding diversity.

## Data Availability

The OTU table (11-packaged/fecal/100 nt/ag_fecal.biom) and human variables (11-packaged/fecal/100 nt/ag_fecal.txt) that support the findings of this study are available at https://github.com/biocore/American-Gut. Python codes on the model analyses are available from the corresponding author upon reasonable request.

## Abbreviations

CD: Cardiovascular disease
SIBO: Small intestinal bacterial overgrowth
MD: Mental disorders
LI: Lactose intolerance
DI: Diabetes
IBD: Inflammatory bowel disease
IBS: Irritable bowel syndrome
CDI: *C. difficile* infection
UH: Unhealthy
OTUs: Operational taxonomic units
AGP: American gut project
ML: Machine learning
Meta: Human variables only
OTUab: OTU abundance only
OTUoc: OTU occurrence only
Meta-OTUab: Both human variable data and OTU abundance
Meta-OTUoc: Both human variable data and OTU occurrence
RF: Random Forest
GBDT: Gradient Boosting Decision Tree
LR: Logistic Regression
XGBoost: eXtreme Gradient Boosting
