# Supplementary material for: Determine independent gut microbiota-diseases association by eliminating the effects of human lifestyle factors
Source: BMC Microbiol. 2022 Jan 3;22:4. doi: 10.1186/s12866-021-02414-9 (PMC8722223; doi:10.1186/s12866-021-02414-9)
Supplement: Supplementary file 1 — Additional file 1: Fig. S1. ROC curves for a selection of the best classification models of eight diseases. Fig. S2. Comparing the differences of AUCs of nine diseases using five feature types after removing probiotics, vitamin B, and vitamin D. Fig. S3. Comparing the differences of AUCs of nine diseases using five feature types after adding gut microbial diversity. Table S1. Frequency of diet and lifestyle factors. Table S2. Basic information of the dataset. Table S3. Comparing AUC values of nine diseases using five feature types. Table S4. AUCs, sensitivity, and specificity for five types of features of the best model selected according to the AUC score. Table S5. Comparing AUC values of nine diseases using five feature types after removing probiotics, vitamin B, and vitamin D. Table S6. Top 10 features after removing probiotics, vitamin B, and vitamin D for all diseases. Table S7. Spearman’s correlations of the human variables and disease. Table S8. Comparing AUC values of nine diseases using five feature types with adding diversity. [file 12866_2021_2414_MOESM1_ESM.zip › Supplementary file.docx]

**Determine independent gut microbiota-diseases association by eliminating the effects of human lifestyle factors**

Congmin Zhu^a,b,c#^, Xin Wang^d^, Jianchu Li^d^, Rui Jiang^e^, Hui Chen^a^, Ting Chen^c*^ and Yuqing Yang^f*^

# Supplementary figures

## Fig. S1 ROC curves for a selection of the best classification models of eight diseases


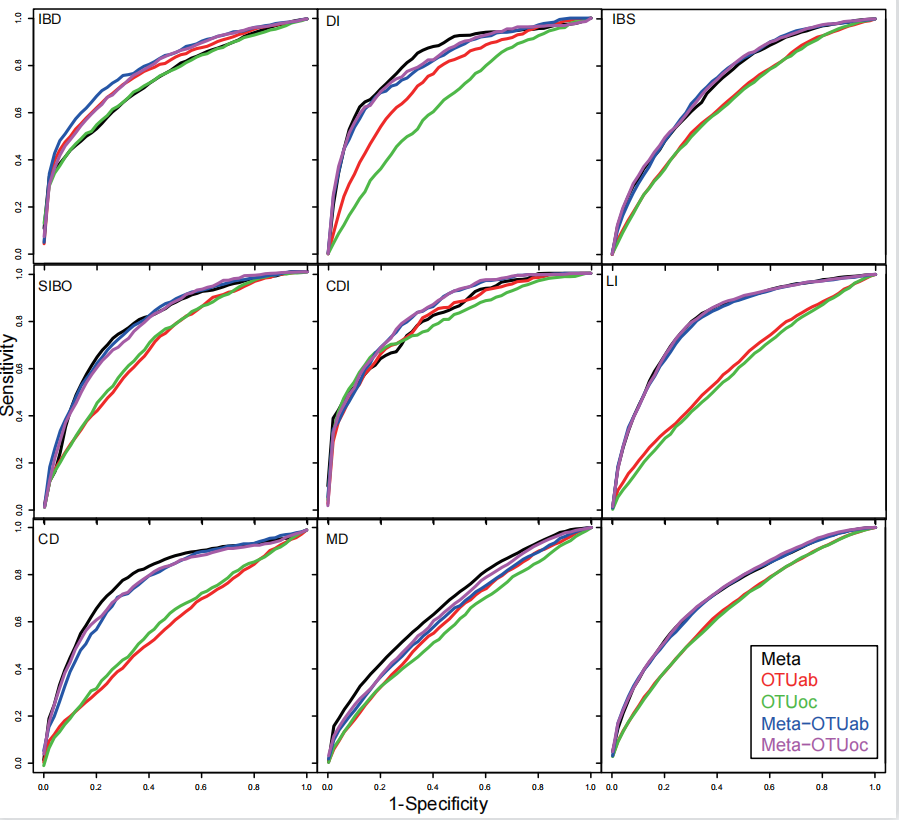


## Fig. S2 Comparing the differences of AUCs of nine diseases using five feature types
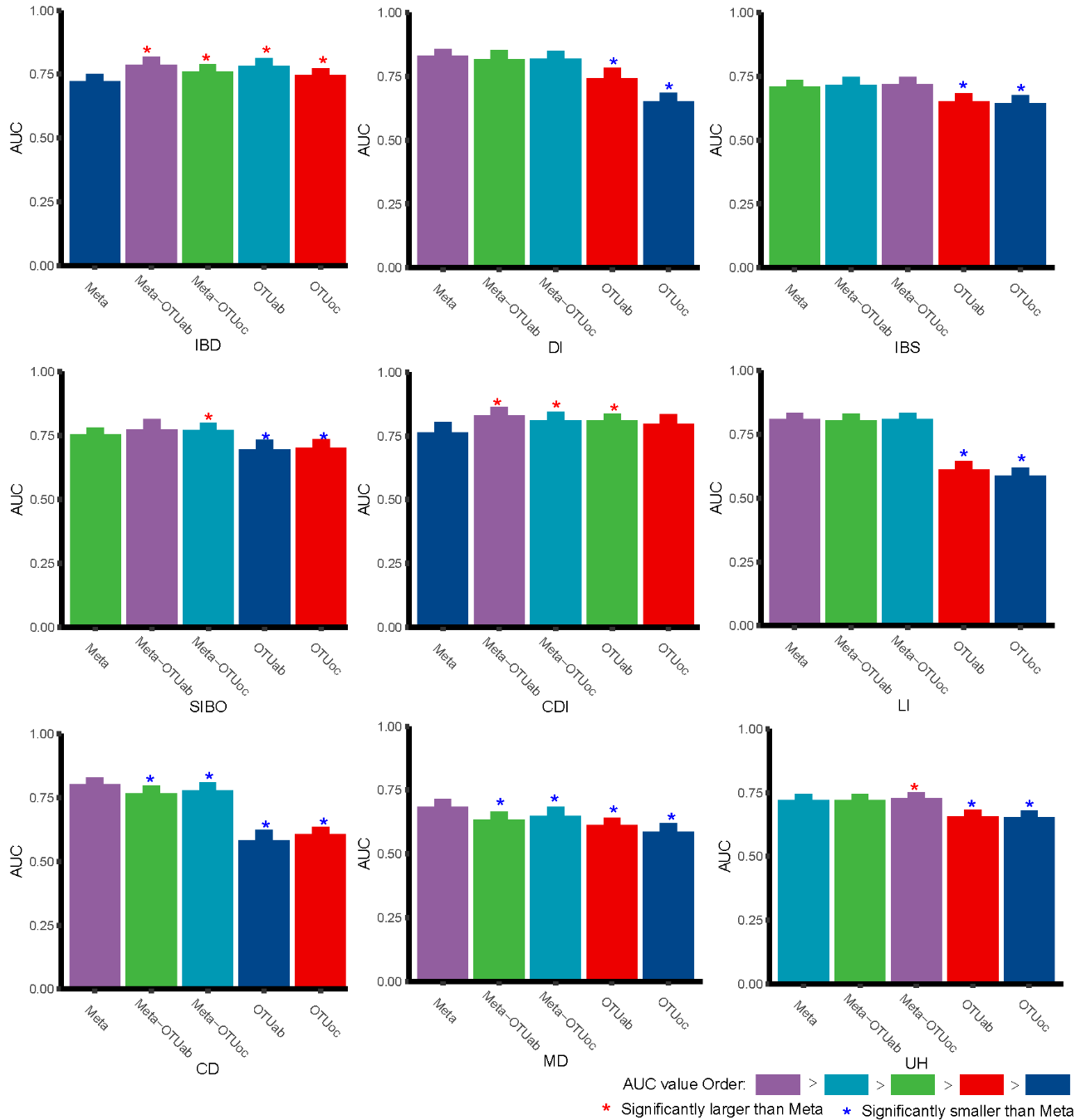
after removing probiotics, vitamin B, and vitamin D.

## Fig. S3 Comparing the differences of AUCs of nine diseases using five feature types
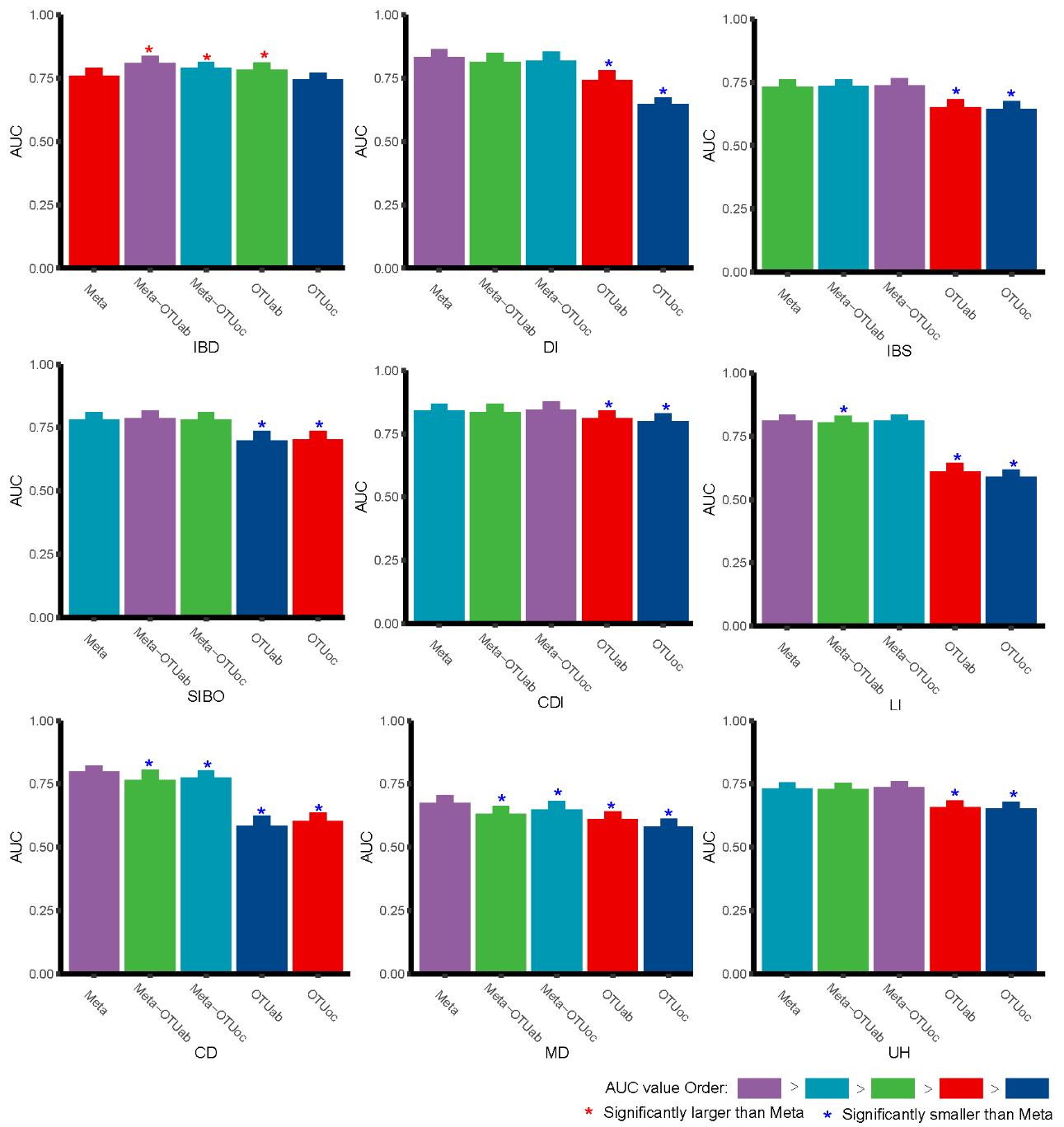
after adding gut microbial diversity.

# Supplementary tables

## Table S1. Frequency of diet and lifestyle factors.

|  | Never | Rarely | Occasionally | Regularly | Daily |
| --- | --- | --- | --- | --- | --- |
| ALCOHOL_FREQUENCY | 1638(21.65%) | 1891(25.00%) | 1792(23.69%) | 1454(19.22%) | 790(10.44%) |
| POULTRY_FREQUENCY | 834(11.02%) | 929(12.28%) | 3028(40.03%) | 2529(33.43%) | 245(3.24%) |
| EXERCISE_FREQUENCY | 211(2.79%) | 810(10.71%) | 1934(25.57%) | 2996(39.60%) | 1614(21.34%) |
| FERMENTED_PLANT_FREQUENCY | 2498(33.02%) | 2577(34.06%) | 1134(14.99%) | 800(10.58%) | 556(7.35%) |
| FROZEN_DESSERT_FREQUENCY | 2128(28.13%) | 3764(49.76%) | 1204(15.92%) | 418(5.53%) | 51(0.67%) |
| FRUIT_FREQUENCY | 378(5.00%) | 995(13.15%) | 1539(20.34%) | 2333(30.84%) | 2320(30.67%) |
| HIGH_FAT_RED_MEAT_FREQUENCY | 1601(21.16%) | 3032(40.08%) | 2010(26.57%) | 830(10.97%) | 92(1.22%) |
| HOMECOOKED_MEALS_FREQUENCY | 122(1.61%) | 197(2.60%) | 464(6.13%) | 2560(33.84%) | 4222(55.81%) |
| RED_MEAT_FREQUENCY | 1127(14.90%) | 1823(24.10%) | 2679(35.41%) | 1777(23.49%) | 159(2.10%) |
| MEAT_EGGS_FREQUENCY | 297(3.93%) | 385(5.09%) | 1182(15.62%) | 3068(40.56%) | 2633(34.81%) |
| MILK_SUBSTITUTE_FREQUENCY | 3586(47.40%) | 1246(16.47%) | 818(10.81%) | 794(10.50%) | 1121(14.82%) |
| MILK_CHEESE_FREQUENCY | 1110(14.67%) | 1323(17.49%) | 1424(18.82%) | 1983(26.21%) | 1725(22.80%) |
| OLIVE_OIL | 467(6.17%) | 719(9.50%) | 1555(20.56%) | 2869(37.92%) | 1955(25.84%) |
| PROBIOTIC_FREQUENCY | 3149(41.63%) | 1498(19.80%) | 728(9.62%) | 760(10.05%) | 1430(18.90%) |
| SALTED_SNACKS_FREQUENCY | 1239(16.38%) | 2724(36.01%) | 2084(27.55%) | 1194(15.78%) | 324(4.28%) |
| SEAFOOD_FREQUENCY | 906(11.98%) | 2256(29.82%) | 3331(44.03%) | 1006(13.30%) | 66(0.87%) |
| SMOKING_FREQUENCY | 7101(93.87%) | 266(3.52%) | 58(0.77%) | 37(0.49%) | 103(1.36%) |
| VEGETABLE_FREQUENCY | 52(0.69%) | 203(2.68%) | 672(8.88%) | 2658(35.14%) | 3980(52.61%) |
| VITAMIN_D_SUPPLEMENT_FREQUENCY | 3322(43.91%) | 930(12.29%) | 536(7.09%) | 775(10.24%) | 2002(26.46%) |
| VITAMIN_B_SUPPLEMENT_FREQUENCY | 4301(56.85%) | 905(11.96%) | 472(6.24%) | 544(7.19%) | 1343(17.75%) |
| WHOLE_GRAIN_FREQUENCY | 943(12.47%) | 1520(20.09%) | 1668(22.05%) | 2101(27.77%) | 1333(17.62%) |
| WHOLE_EGGS | 603(7.97%) | 1203(15.90%) | 2915(38.53%) | 2008(26.54%) | 836(11.05%) |

Rarely: less than once/week; Occasionally: 1–2 times/week; Regularly: 3–5 times/week.

## Table S2. Basic information of the dataset.

|  | Female | Male | Total |
| --- | --- | --- | --- |
| Number | 3982 | 3583 | 7565 |
| AGE_CORRECTED | 47(35, 59) | 46(33, 61) | 47(34, 59) |
| BMI | 22.41(20.28, 25.65) | 24.25(22.04, 26.62) | 23.36(20.96, 26.30) |
| HEIGHT_CM | 165(160, 170) | 178(172, 183) | 170(162, 178) |
| WEIGHT_KG | 61.0(55.00, 70.00) | 78.0(69, 87) | 69(58, 81) |
| LATITUDE | 40.8(34.10, 51.30) | 40.1(33, 50.30) | 40.7(33.60, 51) |
| ELEVATION | 81.2(28.40, 193.07) | 96.1(30.90, 205.35) | 88.0(29.70, 200.60) |
| Caucasian | 3576 | 3181 | 6757 |
| Asian or Pacific Islander | 162 | 178 | 340 |
| Other | 120 | 106 | 226 |
| African American | 29 | 16 | 45 |
| Hispanic | 95 | 102 | 197 |
| CARDIOVASCULAR_DISEASE | 73 | 179 | 252 |
| SIBO | 206 | 100 | 306 |
| MENTAL_ILLNESS | 407 | 270 | 677 |
| LACTOSE | 707 | 502 | 1209 |
| IBS | 667 | 329 | 996 |
| IBD | 164 | 249 | 413 |
| DIABETES | 75 | 100 | 175 |
| CDIFF | 92 | 87 | 179 |
| Unhealth | 1609 | 1301 | 2910 |

The representation format of the values for age, BMI, height, weight, latitude, and elevation are median (lower quartile and upper quartile).

## Table S3. Comparing AUC values of nine diseases using five feature types.

| Feature type | | Meta | OTUab | OTUoc | Meta-OTUab | Meta-OTUoc |
| --- | --- | --- | --- | --- | --- | --- |
| IBD | AUC | 0.74677±0.01240 | 0.78455±0.00905 | 0.74341±0.00696 | 0.80844±0.00855 | 0.79028±0.00637 |
|  | p-value | - | 0.00012 | 0.48579 | <0.00001 | <0.00001 |
| DI | AUC | 0.83022±0.00872 | 0.74401±0.01874 | 0.64577±0.01583 | 0.81209±0.01598 | 0.81621±0.01376 |
|  | p-value | - | <0.00001 | <0.00001 | 0.02024 | 0.03214 |
| IBS | AUC | 0.72978±0.00552 | 0.65015±0.01262 | 0.64453±0.01086 | 0.73573±0.00669 | 0.73953±0.00580 |
|  | p-value | - | <0.00001 | <0.00001 | 0.00734 | 0.00107 |
| SIBO | AUC | 0.78317±0.00616 | 0.69470±0.01840 | 0.70155±0.01462 | 0.78967±0.00989 | 0.78174±0.01151 |
|  | p-value | - | <0.00001 | <0.00001 | 0.07803 | 0.74965 |
| CDI | AUC | 0.81470±0.01122 | 0.80916±0.00922 | 0.79603±0.01700 | 0.83719±0.01308 | 0.84252±0.00959 |
|  | p-value | - | 0.24377 | 0.02344 | 0.00264 | 0.00003 |
| LI | AUC | 0.81189±0.00386 | 0.61220±0.01117 | 0.58964±0.00987 | 0.80580±0.00595 | 0.81105±0.00375 |
|  | p-value | - | <0.00001 | <0.00001 | 0.00036 | 0.29260 |
| CD | AUC | 0.79756±0.00638 | 0.58849±0.02118 | 0.60585±0.01471 | 0.76811±0.02167 | 0.77445±0.00511 |
|  | p-value | - | <0.00001 | <0.00001 | 0.00477 | 0.00004 |
| MD | AUC | 0.68075±0.00679 | 0.61189±0.00853 | 0.58617±0.01565 | 0.63215±0.01002 | 0.65090±0.01577 |
|  | p-value | - | <0.00001 | <0.00001 | <0.00001 | 0.00133 |
| UH | AUC | 0.73032±0.00346 | 0.65692±0.00602 | 0.65317±0.00531 | 0.73021±0.00375 | 0.73582±0.00280 |
|  | p-value | - | <0.00001 | <0.00001 | 0.94993 | <0.00001 |

## Table S4. AUCs, sensitivity, and specificity for five types of features of the best model selected according to the AUC score.

*See the excel file.*

## Table S5. Comparing AUC values of nine diseases using five feature types after removing probiotics, vitamin B, and vitamin D.

| Feature type | | Meta | OTUab | OTUoc | Meta-OTUab | Meta-OTUoc |
| --- | --- | --- | --- | --- | --- | --- |
| IBD | AUC | 0.72128±0.00960 | 0.78271±0.00902 | 0.74511±0.00629 | 0.78659±0.01088 | 0.76038±0.00749 |
|  | p-value | - | <0.00001 | 0.00009 | <0.00001 | <0.00001 |
| DI | AUC | 0.83012±0.00702 | 0.74173±0.02179 | 0.65272±0.01130 | 0.81548±0.01568 | 0.81820±0.01092 |
|  | p-value | - | <0.00001 | <0.00001 | 0.03903 | 0.01167 |
| IBS | AUC | 0.70991±0.00396 | 0.65067±0.01277 | 0.64454±0.01084 | 0.71536±0.01094 | 0.71887±0.00856 |
|  | p-value | - | <0.00001 | <0.00001 | 0.15956 | 0.00759 |
| SIBO | AUC | 0.75445±0.00595 | 0.69504±0.01858 | 0.70182±0.01404 | 0.77323±0.02234 | 0.77115±0.01000 |
|  | p-value | - | 0.00002 | 0.00001 | 0.01636 | 0.00006 |
| CDI | AUC | 0.76323±0.02033 | 0.81131±0.00659 | 0.79560±0.01864 | 0.83095±0.01099 | 0.81189±0.01197 |
|  | p-value | - | 0.00014 | 0.00214 | 0.00004 | 0.00020 |
| LI | AUC | 0.81004±0.00373 | 0.61236±0.01167 | 0.58844±0.00966 | 0.80422±0.00676 | 0.80992±0.00366 |
|  | p-value | - | <0.00001 | <0.00001 | 0.00388 | 0.84246 |
| CD | AUC | 0.80253±0.00488 | 0.58362±0.01994 | 0.60582±0.00868 | 0.76569±0.01259 | 0.77878±0.00935 |
|  | p-value | - | <0.00001 | <0.00001 | <0.00001 | 0.00011 |
| MD | AUC | 0.68377±0.01071 | 0.61211±0.00813 | 0.58570±0.01490 | 0.63446±0.01111 | 0.64918±0.01484 |
|  | p-value | - | <0.00001 | <0.00001 | <0.00001 | 0.00072 |
| UH | AUC | 0.72088±0.00298 | 0.65693±0.00595 | 0.65317±0.00531 | 0.71932±0.00386 | 0.72656±0.00395 |
|  | p-value | - | <0.00001 | <0.00001 | 0.43482 | 0.00027 |

## Table S6. Top 10 features after removing probiotics, vitamin B, and vitamin D for all diseases.

| IBD | DI | IBS | SIBO | CDI | LI | CD | MD | UH |
| --- | --- | --- | --- | --- | --- | --- | --- | --- |
| Meta-OTUoc | Meta-OTUoc | Meta-OTUab | Meta-OTUab | Meta | Meta |  | Meta-OTUoc | Meta-OTUoc |
| BMI/45.3 | BMI/1.7 | MILK_CHEESE_FREQUENCY/3.4 | MILK_CHEESE_FREQUENCY/10.5 | HEIGHT_CM/1.6 | MILK_CHEESE_FREQUENCY/1.1 | AGE_CORRECTED/1.5 | MILK_SUBSTITUTE_FREQUENCY/5.4 | MILK_CHEESE/1 |
| HEIGHT_CM/48.3 | AGE_CORRECTED/7.2 | female/4.9 | WHOLE_GRAIN_FREQUENCY/16.8 | BMI/5.3 | MILK_SUBSTITUTE_FREQUENCY/1.9 | female/2.2 | AGE_CORRECTED/28.2 | c_Clostridia;o_Clostridiales;f_;g_;s_/7.8 |
| c_Clostridia;o_Clostridiales;f_Ruminococcaceae;g_Ruminococcus;s_/48.4 | c_Clostridia;o_Clostridiales;f_Ruminococcaceae;g_;s_/17.8 | c_Gammaproteobacteria;o_Enterobacteriales;f_Enterobacteriaceae;g_Enterobacter;s_radicincitans/5.3 | FROZEN_DESSERT_FREQUENCY/17 | MILK_CHEESE_FREQUENCY/6.3 | ELEVATION/7.3 | RED_MEAT_FREQUENCY/5.2 | ELEVATION/34.4 | MILK_SUBSTITUTE/10.3 |
| c_Clostridia;o_Clostridiales;f_Ruminococcaceae;g_Ruminococcus;s_/48.8 | WEIGHT_KG/22.6 | AGE_CORRECTED/9.6 | c_Gammaproteobacteria;o_Enterobacteriales;f_Enterobacteriaceae;g_Enterobacter;s_radicincitans/28.7 | FROZEN_DESSERT_FREQUENCY/6.7 | ALCOHOL_FREQUENCY/7.7 | HEIGHT_CM/6.4 | HEIGHT_CM/35.5 | c_Clostridia;o_Clostridiales;f_;g_;s_/15.2 |
| c_Clostridia;o_Clostridiales;f_Lachnospiraceae;g_;s_/50.3 | c_Clostridia;o_Clostridiales;f_Ruminococcaceae;g_Ruminococcus;s_/23.3 | LATITUDE/10.5 | c_Clostridia;o_Clostridiales;f_Ruminococcaceae;g_Oscillospira;s_/32.1 | AGE_CORRECTED/9.5 | HEIGHT_CM/8.5 | WEIGHT_KG/7.5 | BMI/35.9 | c_Clostridia;o_Clostridiales;f_Ruminococcaceae;g_Ruminococcus;s_/15.2 |
| c_Clostridia;o_Clostridiales;f_[Tissierellaceae];g_Anaerococcus;s_/50.4 | ALCOHOL_FREQUENCY/26.7 | SEAFOOD_FREQUENCY/25.4 | c_Clostridia;o_Clostridiales;f_Veillonellaceae;g_Veillonella;s_parvula/44.4 | LATITUDE/10.9 | BMI/8.5 | FRUIT_FREQUENCY/8.6 | ALCOHOL_FREQUENCY/42.3 | FROZEN_DESSERT/19.8 |
| c_Clostridia;o_Clostridiales;f_;g_;s_/54.1 | MILK_CHEESE_FREQUENCY/34.2 | MILK_SUBSTITUTE_FREQUENCY/27.8 | c_Clostridia;o_Clostridiales;f_Ruminococcaceae;g_;s_/49.7 | FERMENTED_PLANT_FREQUENCY/11.4 | LATITUDE/8.6 | OLIVE_OIL/11.6 | WEIGHT_KG/46.7 | c_Clostridia;o_Clostridiales;f_Lachnospiraceae;g_;s_/21.8 |
| c_Clostridia;o_Clostridiales;f_Ruminococcaceae;g_;s_/54.2 | ELEVATION/34.7 | WEIGHT_KG/32.4 | POULTRY_FREQUENCY/55.1 | ALCOHOL_FREQUENCY/11.5 | Caucasian/9 | LATITUDE/11.6 | FROZEN_DESSERT_FREQUENCY/48.9 | AGE_CORRECTED/22.6 |
| c_Clostridia;o_Clostridiales;f_;g_;s_/60.6 | HEIGHT_CM/36.1 | c_Clostridia;o_Clostridiales;f_Ruminococcaceae;g_;s_/40.2 | c_Clostridia;o_Clostridiales;f_Lachnospiraceae;g_[Ruminococcus];s_/55.8 | MILK_SUBSTITUTE_FREQUENCY/13 | AGE_CORRECTED/9.7 | BMI/11.6 | c_Clostridia;o_Clostridiales;f_Clostridiaceae;g_;s_/60.2 | BMI/23.9 |
| MILK_CHEESE/66.3 | c_Erysipelotrichi;o_Erysipelotrichales;f_Erysipelotrichaceae;g_Clostridium;s_saccharogumia/36.9 | HEIGHT_CM/49.9 | c_Clostridia;o_Clostridiales;f_;g_;s_/56.6 | WHOLE_EGGS/13.3 | SALTED_SNACKS_FREQUENCY/10.1 | SEAFOOD_FREQUENCY/13.2 | c_Clostridia;o_Clostridiales;f_Clostridiaceae;g_;s_/63.4 | c_Clostridia;o_Clostridiales;f_Ruminococcaceae;g_;s_/24.9 |

## Table S7. Spearman’s correlations of the human variables and disease.

*See the excel file.*

## Table S8. Comparing AUC values of nine diseases using five feature types with adding diversity.

| Feature type | | Meta | OTUab | OTUoc | Meta-OTUab | Meta-OTUoc |
| --- | --- | --- | --- | --- | --- | --- |
| IBD | AUC | 0.75969±0.00971 | 0.78275±0.00914 | 0.74514±0.00620 | 0.80869±0.00834 | 0.79025±0.00422 |
|  | p-value | - | 0.00059 | 0.00553 | 0.00001 | 0.00001 |
| DI | AUC | 0.83414±0.01077 | 0.74223±0.01926 | 0.64630±0.00781 | 0.81368±0.01608 | 0.81816±0.01526 |
|  | p-value | - | <0.00001 | <0.00001 | 0.00430 | 0.03314 |
| IBS | AUC | 0.73167±0.00992 | 0.65047±0.01268 | 0.64452±0.01086 | 0.73438±0.00707 | 0.73630±0.00843 |
|  | p-value | - | <0.00001 | <0.00001 | 0.43753 | 0.21600 |
| SIBO | AUC | 0.78294±0.00810 | 0.69788±0.01854 | 0.70225±0.01439 | 0.78744±0.01056 | 0.78089±0.00873 |
|  | p-value | - | <0.00001 | <0.00001 | 0.42007 | 0.46580 |
| CDI | AUC | 0.84229±0.00627 | 0.81069±0.01026 | 0.79894±0.01137 | 0.83562±0.01228 | 0.84507±0.01231 |
|  | p-value | - | 0.00001 | 0.00002 | 0.16229 | 0.52936 |
| LI | AUC | 0.81107±0.00378 | 0.61208±0.01144 | 0.58833±0.00968 | 0.80516±0.00543 | 0.81006±0.00386 |
|  | p-value | - | <0.00001 | <0.00001 | 0.00030 | 0.35672 |
| CD | AUC | 0.79758±0.00413 | 0.58281±0.02229 | 0.60305±0.01198 | 0.76549±0.01959 | 0.77528±0.00688 |
|  | p-value | - | <0.00001 | <0.00001 | 0.00070 | 0.00004 |
| MD | AUC | 0.67532±0.01000 | 0.60984±0.01062 | 0.58073±0.01176 | 0.63243±0.01043 | 0.64887±0.01313 |
|  | p-value | - | <0.00001 | <0.00001 | <0.00001 | 0.00088 |
| UH | AUC | 0.73188±0.00249 | 0.65689±0.00597 | 0.65317±0.00531 | 0.73060±0.00359 | 0.73569±0.00422 |
|  | p-value | - | <0.00001 | <0.00001 | 0.44644 | 0.00568 |
